# Management of a Submandibular Sialolith: A Case Report

**DOI:** 10.7759/cureus.61812

**Published:** 2024-06-06

**Authors:** Arshjot S Basra, Swapnil Mohod, Sourabh B Shinde, Lavannya D Phaye, Prachi Khandelwal

**Affiliations:** 1 Oral Medicine and Radiology, Sharad Pawar Dental College and Hospital, Datta Meghe Institute of Higher Education and Research, Wardha, IND; 2 Periodontics and Implantology, Sharad Pawar Dental College and Hospital, Datta Meghe Institute of Higher Education and Research, Wardha, IND; 3 Dentistry, Sharad Pawar Dental College and Hospital, Datta Meghe Institute of Higher Education and Research, Wardha, IND

**Keywords:** difficulty in deglutition, minimal invasive approach, extracorporeal shock wave lithotripsy, sialadenectomy, sialolith

## Abstract

Sialolithiasis is a condition that is characterized by the obstruction of the salivary gland duct opening by calcified mineral deposits due to various factors discussed in this case report. The most common symptom associated with the pathology is difficulty in deglutition, which can often lead to dehydration due to poor water intake. This, in turn further increases the viscosity of saliva which further promotes the formation of sialoliths. The management is dictated by the location and size of the sialolith, and in this case report, the significance of conservative treatment is emphasized while acknowledging the importance of invasive treatment when necessary.

## Introduction

Sialolithiasis is the most common disease of the salivary glands and is characterized by the formation of salivary stones (sialoliths). A range of symptoms, including swelling in the affected gland, difficulty while eating, and pain, especially during meals when saliva production increases, can be the presenting feature because these stones obstruct the flow of saliva [1]. It is perplexing to note that the submandibular gland is where the majority of this disease usually manifests itself, making up between 80.0% and 92.0%, followed by the parotid gland with 5.0%-20.0%, and the sublingual gland with 1.0%-2.0% of cases [2,3].

Various descriptions have been put forward to explain this predilection and they are as follows: (A) Anatomy: the submandibular duct has a greater length and angle than other excretory ducts in the salivary gland. This extended dimensionality can create retention zones for saliva and promote the accumulation of crystals, hence facilitating lithogenesis [4]; (B) Composition of saliva: the submandibular duct contributes more minerals like calcium than any other component. Since more salts are present in its secretion, crystallization occurs here frequently. The occurrence of sialomicroliths, which are observed in the normal salivary glands of symptomless individuals, could have a role in the etiology [5]; (C) Dehydration: the increased salivary thickness caused by dehydration also contributes to the formation of calculus [6].

Several factors may increase an individual's risk of developing sialolithiasis, which include age (mostly adults between 30 and 60 years are affected by sialoliths); sex (males are more prone to develop sialoliths than females); medical conditions (such as Sjögren's syndrome, an autoimmune disease affecting saliva production) and recurrent parotid infections, can increase the risk); and medications which contribute to dehydration or alter salivary composition, potentially increasing the risk of sialolith formation [7].

Diagnosing submandibular sialolithiasis is typically done through a combination of imaging studies and clinical examination. During the clinical examination, the healthcare provider may observe swelling and tenderness in the affected area. Imaging techniques can be used in the visualization of the sialolith, including sialography (which involves taking an X-ray after injecting a contrast dye into the salivary duct), CT scans, and ultrasonography. These diagnostic methods help determine the size and location of the sialolith. Submandibular sialolithiasis is a condition characterized by the presence of stones (sialoliths) in the submandibular salivary gland. It can cause various signs and symptoms, such as pain or discomfort in the floor of the mouth or under the jaw, swelling in the affected area, difficulty opening the mouth or swallowing, dryness, or an altered taste in the mouth.

The choice of treatment for submandibular sialolithiasis differs due to factors like the size and location of the calcification. For smaller stones, healthcare providers may initially recommend non-invasive approaches to promote stone passage, such as sialagogues (medications that stimulate saliva production), warm compresses (applying warm compresses to the affected area), and massage (gently massaging the area to help dislodge the stone [7]. If conservative measures are ineffective or for larger stones, minimally invasive procedures may be considered, such as sialendoscopy (using a small camera to visualize and potentially remove the stone) and laser lithotripsy (fragmenting the stone using laser energy) [7]. In rare cases where other treatments are not viable or successful, surgical removal of the submandibular gland may be essential. However, there seems to be a lack of documentation on the conservative treatment approach, even though the surgical treatment of sialolithiasis is fairly well documented.

## Case presentation

A 21-year-old male patient reported to the Department of Oral Medicine and Radiology, Datta Meghe Institute of Higher Education and Research, Wardha, India, with the chief complaint of pain below the tongue on the left side of the jaw for three days. The pain was sudden in onset and sharp shooting in nature, which aggravated during movements of the lower jaw, especially during mastication and prolonged speech, and was relieved on resting the jaw. There was no significant medical or dental history. The patient had a history of cigarette smoking for the past three years and, on average, smoked five to six cigarettes per day.

**Figure 1:**
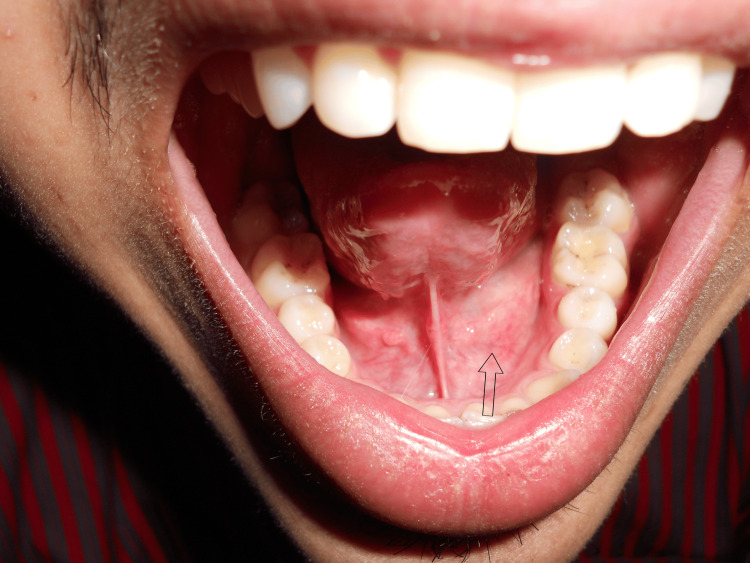
There is swelling in the floor of the mouth, lateral to the lingual frenum.

On extraoral examination, the facial profile was bilaterally symmetrical. During palpation, the left submandibular gland was tender and firm. On intraoral examination, the left submandibular duct opening was inflamed; however, notably, erythema was not seen in relation to the ductal opening. No associated bleeding or pus discharge was observed (Figure 1).

**Figure 2:**
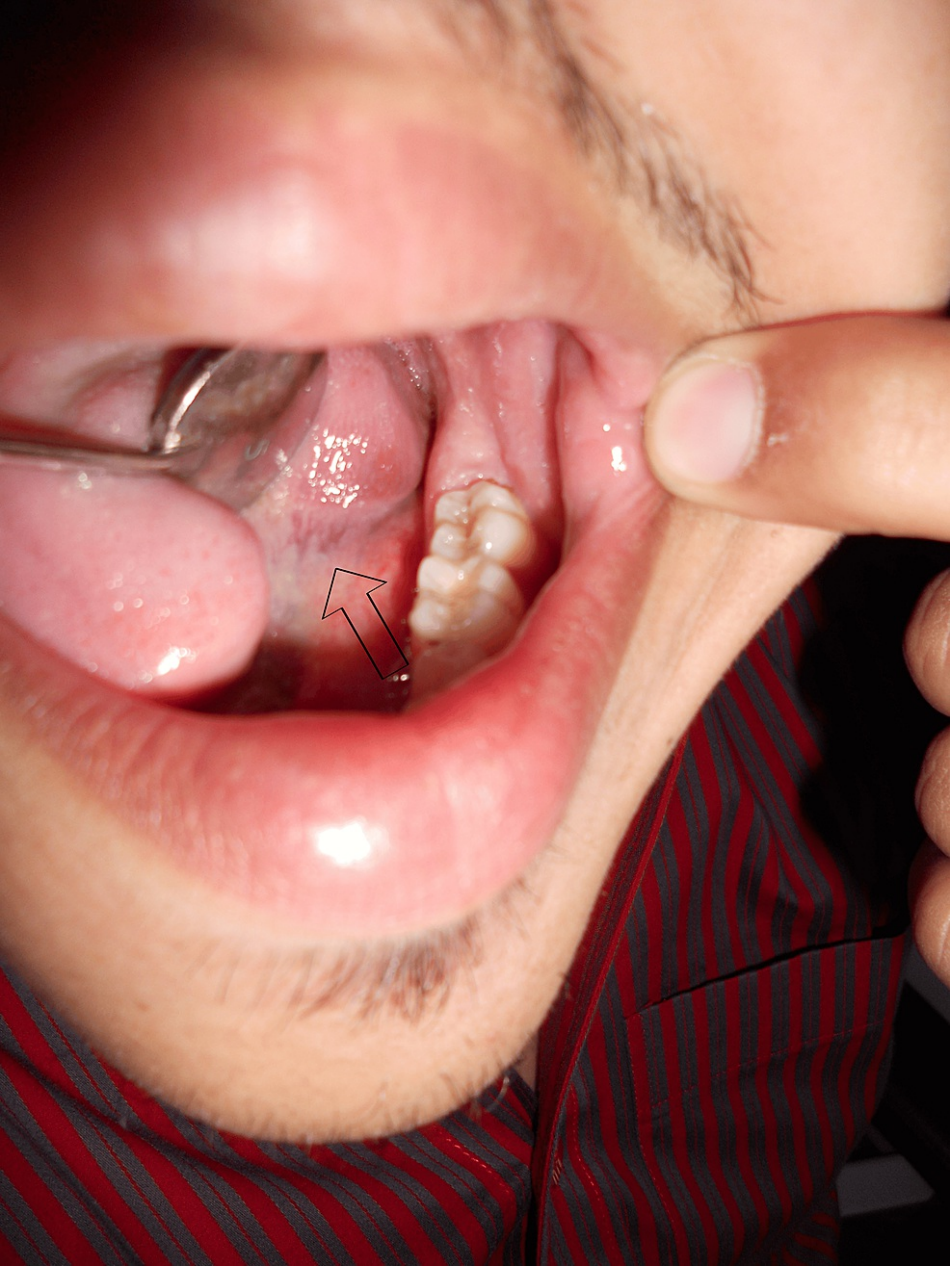
Erythema along the Wharton duct is seen.

The swelling extended from the base of the lingual frenum to the base of the tongue anterior-posteriorly; moreover, on palpation, the swelling was firm in consistency. On examining the left lateral aspect of the tongue, swelling associated with distinguished erythema medial to tooth numbers 36 and 37 along the Wharton duct was reported (Figure 2). Based on clinical features, the provisional diagnosis was submandibular sialolith, and the differential diagnosis was phlebolith, submandibular gland infection, and hemangioma with calcification.

**Figure 3:**
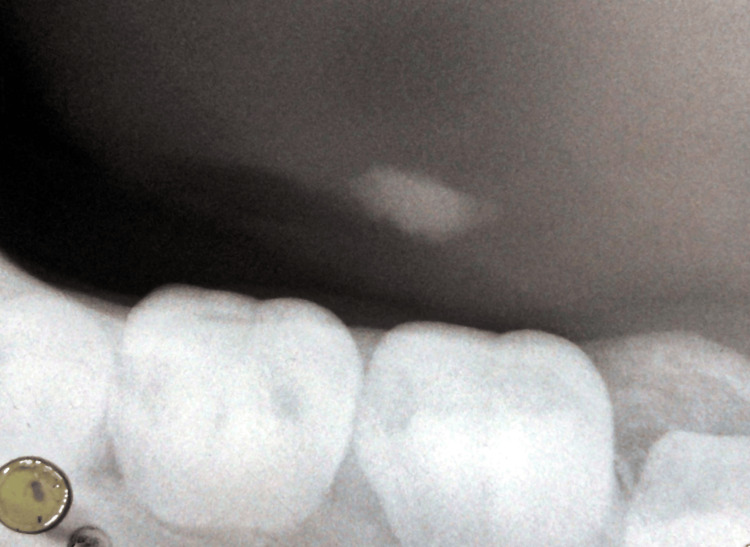
A mandibular occlusal radiograph shows radiopacity medial to teeth numbers 36 and 37.

An occlusal radiograph of tooth numbers 36 and 37 was taken. On the radiograph, radiopacity extending from the distal cusp of 36 to the mesial cusp of 37 was seen (Figure 3). Based on radiographic and clinical findings, the case was diagnosed as left submandibular sialolithiasis.

The treatment plan was dictated by the size and location of the sialolith. Firstly, due to the small size of the sialolith, it was judicious to implement a conservative approach, which included warm compression (for five minutes, four times daily for seven days) and massage (for 10 minutes, three times daily for seven days), increasing water intake, and instructing the patient to quit smoking. Along with these measures, the patient was prescribed sialagogues and antibiotics (xylitol 200 mg, three times daily for seven days, and amoxicillin 500 mg, three times daily for five days), and bimanual palpation was performed and explained to the patient as it can be helpful to remove small sialoliths through the duct opening. The patient was made aware of the next line of treatment in case a conservative approach is unsuccessful, which includes sialendoscopy, extracorporeal shock wave lithotripsy (ESWL), and surgical removal. The patient was recalled after seven days for reevaluation.

**Figure 4:**
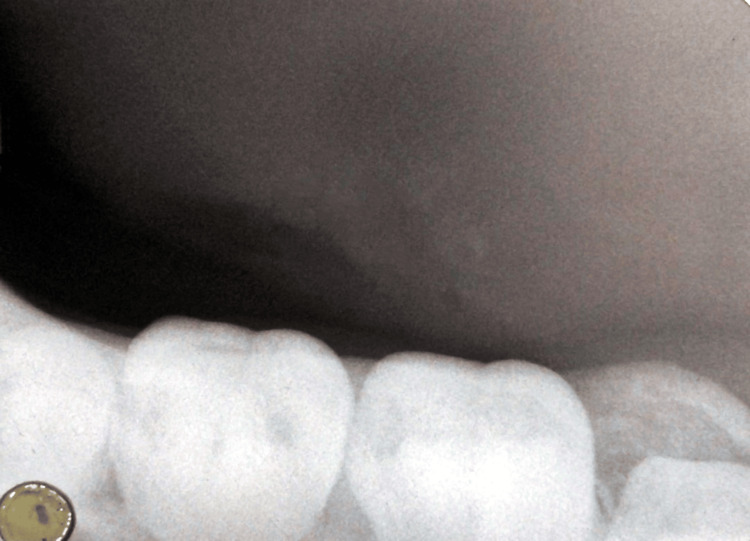
Occlusal radiograph obtained on the recall visit

On the recall visit, the patient reported that he had not smoked since the last visit seven days ago and was able to comfortably eat food without experiencing any pain. The facial profile was bilaterally symmetrical, and on bimanual palpation, submandibular glands on both sides were non-tender. The patient reported that he was not aware of any solids coming out while eating or drinking. On intraoral examination, swelling on the left lateral border of the tongue, which was previously present, has subsided, and no pus or blood discharge was observed. On taking an occlusal radiograph, sialolith was no longer seen in the imaging (Figure 4). 

The patient was advised to avoid smoking, drink three to four liters of water every day, especially during the summer, and maintain good oral hygiene to prevent sialolith formation as much as possible.

## Discussion

Sialolithiasis, also known as salivary stones or calculi, is a condition characterized by the formation of stones within the salivary glands or their ducts. These stones, typically composed of calcium phosphate and hydroxyapatite, can obstruct salivary flow and lead to a cascade of signs and symptoms [7, 8].

The exact cause of sialolithiasis is not fully understood, but numerous factors are believed to contribute which are (A) Dehydration: it can lead to concentrated saliva, making it more susceptible to mineral precipitation and stone formation [9]; (B) Diets high in calcium, oxalates, and certain medications: those can alter the salivary composition and increase the risk of stones [9]; (C) Sialadenitis (inflammation of the salivary gland): previous episodes of salivary gland inflammation can damage tissues and predispose to stone formation [10]; (D) Anatomical factors: narrowing of salivary ducts due to strictures or enlarged lymph nodes can create stagnation of saliva and promote stone formation; (E) Age and sex: these are more common in adults aged 30-60 and affects males slightly more often than females [7]; (F) Medical conditions: conditions like Sjögren's syndrome (autoimmune disease affecting saliva production) and recurrent parotid infections can increase the risk [9].

The most common symptoms of sialolithiasis are unilateral swelling and pain in the affected salivary gland. This pain typically worsens during meals as the stone obstructs saliva production and increases flow. Other symptoms may include dry mouth, difficulty swallowing, pus discharge from the salivary duct opening, facial redness and tenderness, and fever (if an infection develops) [7,8]. The location of the stone can influence the presenting symptoms. Stones in the submandibular gland (Wharton's duct) are the most common and cause swelling below the jaw and near the angle of the mandible [9]. Parotid gland stones (Stensen's duct) cause swelling in the cheek area, and sublingual gland stones are less frequent but can cause swelling under the tongue [7].

Treatment for sialolithiasis

Conservative Management

Treatment depends on the severity of symptoms and the size and location of the stone. The treatment methods generally include (A) warm compresses and massages, which can help promote salivary flow and dislodge small stones; (B) increased hydration to dilute saliva and ease the passage of the stone; (C) sialagogues to stimulate saliva production to help expel the stone; (D) antibiotics to control infection, if present [6, 7].

Minimally invasive techniques may be attempted for stones lodged near the duct opening, such as a sialendoscopy, where a thin, flexible scope is inserted into the salivary duct to visualize and possibly remove the stone with laser fragmentation or basket retrieval, or ESWL, where shock waves are used to break down the stone into smaller fragments that can pass through the duct easily [7-10]. If conservative and minimally invasive methods fail, surgery may be necessary to remove the stone or, in rare cases, the affected salivary gland as a final resort.

Surgical Management

Surgical removal of salivary gland stones, also known as sialadenectomy or occasionally gland excision, is a last resort procedure typically reserved for situations where conservative and minimally invasive techniques have been unfruitful. It's important to note that surgeons aim to preserve the salivary gland whenever possible, as these glands play a critical role in digestion and maintaining oral health. Below is a detailed breakdown of the surgical removal process, referencing relevant sources.

Preoperative evaluation: A thorough medical history-taking and physical examination are conducted to assess overall health and fitness for surgery. Imaging studies like X-rays, CT scans, or sialography (a special X-ray with contrast dye injected into the salivary duct) are used to pinpoint the stone's location and assess the anatomy of the gland and surrounding structures. Blood tests may be ordered to evaluate general health and clotting function [7-9].

Surgical procedure: The type of anesthesia used depends on the complexity of the case and the surgeon's preference. It can range from local anesthesia with sedation to general anesthesia [11].

Approaches: There are two main surgical approaches for removing salivary gland stones, depending on the location of the stone and gland involved. The transoral approach involves making an incision inside the mouth, directly over the affected salivary duct. This approach is preferred for stones located near the duct opening in the submandibular or sublingual glands [7]. The extraoral approach involves making an incision on the skin, either in the cheek area for parotid gland stones or below the jawline for submandibular gland stones. This approach is necessary for deeper stones or when the transoral approach is not feasible [7, 11]. Once the surgeon has accessed the salivary gland and duct, they will locate the stone.

Techniques for stone removal may involve direct visualization and extraction for larger stones, where the surgeon may be able to visualize and remove the stone using surgical instruments directly. Another technique is sialendoscopy, where, in some cases, a sialendoscope, a thin, flexible scope with a camera, will be inserted into the duct to visualize and potentially fragment the stone with lasers or remove it with basket retrieval tools [7, 8]. Gland excision, if necessary, may be performed in rare cases. If the stone is deeply embedded within the gland, severely damaged tissue is present, or recurrent stone formation is a concern, the surgeon may opt to remove the entire affected salivary gland [7,8,11]. The surgical site is then irrigated and meticulously closed with sutures. Depending on the approach, the sutures may be dissolvable or require removal at a follow-up appointment [7,11].

Postoperative care: Pain medication is typically prescribed to manage discomfort. Warm compresses on the surgical site may be recommended to promote healing and reduce swelling. A soft diet is often advised for initial recovery to minimize strain on the surgical site. Antibiotics may be prescribed to prevent infection. Oral hygiene practices are crucial to prevent complications [7,9].

The differential diagnosis includes a radiolucent phlebolith, infections, inflammatory conditions (radiotherapy reaction, sarcoidosis, Sjögren's syndrome), palatine tonsilliths (punctuate and multiple), dystrophic calcification of lymph nodes, masses (neoplastic and nonneoplastic), and hemangiomas with calcification [12, 13]. Following removal, medical professionals should counsel patients to follow a diet high in liquids, proteins, and acidic foods in order to prevent new sialoliths from forming in the salivary gland [12].

## Conclusions

Sialolithiasis is a common cause of swelling in the salivary gland and pain. Early diagnosis and prompt intervention are crucial to prevent complications like infection and abscess formation. Conservative treatment spares the patient from the agony, stress, and postoperative pain associated with invasive procedures like surgical removal. While conservative management is often successful, minimally invasive techniques and surgery offer effective options for more stubborn stones. However, the case selection for either treatment approach is crucial to providing quality treatment and, at the same time, preventing any unnecessary invasive procedure.
